# robin2: accelerating single-cell data clustering evaluation

**DOI:** 10.1093/bioadv/vbaf184

**Published:** 2025-08-06

**Authors:** Valeria Policastro, Dario Righelli, Luisa Cutillo, Annamaria Carissimo

**Affiliations:** Department of Political Science, University of Naples Federico II, 80133 Naples, Italy; Istituto per le Applicazioni del Calcolo “Mauro Picone”, Consiglio Nazionale delle Ricerche (CNR), 80131 Naples, Italy; Department of Electrical Engineering and Information Technology, University of Naples Federico II, 80125 Naples, Italy; School of Mathematics, University of Leeds, LS2 9JT Leeds, United Kingdom; Istituto per le Applicazioni del Calcolo “Mauro Picone”, Consiglio Nazionale delle Ricerche (CNR), 80131 Naples, Italy

## Abstract

**Motivation:**

The rapid expansion of single-cell RNA sequencing (scRNA-seq) technologies has increased the need for robust and scalable clustering evaluation methods. To address these challenges, we developed robin2, an optimized version of our R package robin. It introduces enhanced computational efficiency, support for high-dimensional datasets, and harmonious integration with R’s base functionalities for robust network analysis.

**Results:**

robin2 offers improved functionality for clustering stability validation and enables systematic evaluation of community detection algorithms across various resolutions and pipelines. The application to Tabula Muris and PBMC scRNA-seq datasets confirmed its ability to identify biologically meaningful cell subpopulations with high statistical significance. The new version reduces computational time by 9-fold on large-scale datasets using parallel processing.

**Availability and implementation:**

The robin2 package is freely available on CRAN at https://CRAN.R-project.org/package=robin. Comprehensive documentation and a detailed analysis vignette are available on GitHub at https://drighelli.github.io/scrobinv2/index.html.

## 1 Introduction

Biological systems are often represented as networks to model the complex interactions between their components. Mathematically, a network can be defined as a graph G=(V,E), where *V* is the set of nodes (e.g. genes, proteins, or cells) and *E* represents the edges that encode relationships between the nodes. In the context of single-cell transcriptomics (scRNA-seq), graphs are particularly useful for representing cells as nodes and their transcriptional similarities as edges.

One of the main goals of scRNA-seq is to identify distinct cell subpopulations based on their transcriptional profiles. This task is typically addressed using unsupervised clustering, which groups cells with similar gene expression patterns. Clustering methods such as k-means and hierarchical clustering, although widely used, often face computational limitations when applied to large scRNA-seq datasets. In contrast, graph-based clustering methods, such as community detection algorithms, offer a more scalable and efficient solution for clustering millions of cells. A significant challenge remains: determining how accurately clusters derived from scRNA-seq data reflect true biological cell subtypes. Without a reliable ground truth, evaluating the accuracy of clustering results becomes difficult.

In network science, this translates into validation of the results produced by community detection algorithms. The primary challenge lies in determining whether the detected communities are significant or simply artifacts of random edge placement within the network. In our previous paper (Carissimo *et al.* 2018), we addressed this issue, showing that if a partition is significant, it will be recovered even if the graph structure is modified. In contrast, if a partition is not significant, a minimal modification of the graph will be sufficient to change the partition. This basic idea is then expanded and implemented in our R package robin (ROBustness In Network) (Policastro *et al.* 2021) where we evaluate the robustness of the community structure of a network found by one or more methods to give indications about their reliability.

Although our approach can be applied to any type of graph in biological, sociological, and physical fields, in this article, we present robin2, an updated version of robin, in which the method has been optimized to better accommodate large dataset as scRNA-seq data, with a focus on enhancing speed and efficiency to address the unique characteristics of these datasets.

## 2 Materials and methods

### 2.1 robin

The robin R package (Policastro *et al.* 2021) is a tool for evaluating the robustness and reliability of community detection algorithms applied to networks. Its methodology comprises two core procedures. The first (robinCompare) compares two algorithms to determine which is better suited for the given network, producing stability measure curves for each. The second (robinRobust) assesses the stability of a single algorithm under random perturbations of the network structure by comparing stability metrics from the original network to those from a null model. Both these analyses leverage functional statistical tests, including Gaussian Process Regression and the Interval Testing Procedure, to assess statistical significance and highlight key differences.

robin offers users flexibility through customizable stability metrics, perturbation strategies, and null models. It integrates seamlessly with *igraph* algorithms and includes visual tools for analysis and interpretation of results. To evaluate stability, robin uses network partition similarity measures. Its perturbation strategy introduces controlled variability by rewiring edges while preserving the original number of nodes and edges. The perturbation levels typically range from zero to a maximum of 60%, beyond which the network becomes random. Two statistical tests are used to validate the robustness of community detection algorithms by comparing stability measure curves, Gaussian Process Regression (GP) and the Interval Testing Procedure (ITP) (Policastro *et al.* 2021).

### 2.2 robin2 for clustering validation in single cell

For scRNA-seq data analysis, several statistical techniques have been developed and implemented (Trapnell *et al.* 2014, Satija *et al.* 2015, Wolf *et al.* 2018, Amezquita *et al.* 2020). While each of these pipelines differ in parameters and specific analytical approaches, they share a set of fundamental steps (Slovin *et al.* 2021).

First, data normalization is crucial to remove non-biological variations and make gene expression comparable across individual cells. This is typically achieved by dividing the gene counts by each cell’s total number of reads.

Next, feature selection is performed to identify genes with relevant biological information while excluding non-informative ones. Typically, this involves selecting a subset of highly variable genes based on metrics such as variance or coefficient of variation. Following this, a linear dimensional reduction technique like principal component analysis (PCA) is performed to condense the complexity of the data into a lower-dimensional space, focusing on extracting principal components allowing to explain data variability by construction. For better interpretability of such high-dimensionality data, visualization is usually accomplished through nonlinear dimension reduction techniques like t-SNE and UMAP, which effectively projects the data into a lower-dimensional space suitable for interpretation.

Finally, clustering plays a fundamental role in identifying cell subpopulations by grouping cells based on their transcriptional similarities. These clusters often reflect distinct cell types, as transcriptional patterns are typically indicative of cellular identity and function. This approach enables the discovery of novel cell types and the characterization of distinct subpopulations within a single-cell state. Several unsupervised clustering methods have been used after feature selection and data dimensionality reduction to partition single-cell data. These include techniques such as k-means, hierarchical clustering, and graph-based approaches. Graph-based clustering methods, specifically, stand out for their scalability, making them particularly well-suited for large-scale datasets. These approaches can efficiently handle graphs representing hundreds of thousands or even millions of cells, providing robust solutions for analyzing high-dimensional single-cell data. A correct identification of cell types is the most important part of a single-cell analysis, with this work our aim is to define which is the clustering method that better approximates cell subtypes.

A graph G=(V,E) is constructed, where the nodes *V* represent individual cells, and the edges *E* capture the degree of similarity between cell pairs. Typically, this graph is built using the K-Nearest Neighbors (KNN) algorithm applied to the principal component (PC) reduced space, where each cell is connected to its *K* most similar neighbors. To enhance the graph’s representation, edge weights are refined using Jaccard similarity, which quantifies the proportion of shared neighbors between two cells.

Once the graph is constructed, a community detection algorithm, such as the widely used Louvain method, is applied to identify cell clusters. These clusters are assumed to correspond to distinct cell subtypes. In the absence of a definitive ground truth, our goal is to assess how accurately these clusters represent distinct cell subtypes, offering biologically meaningful insights. To address this issue, we significantly improved the two procedures previously developed in robin (Policastro *et al.* 2021). These improvements greatly boosted the performance of the method, enabling its application to very large networks, such as those encountered in single-cell data analysis.

**Figure 1.**
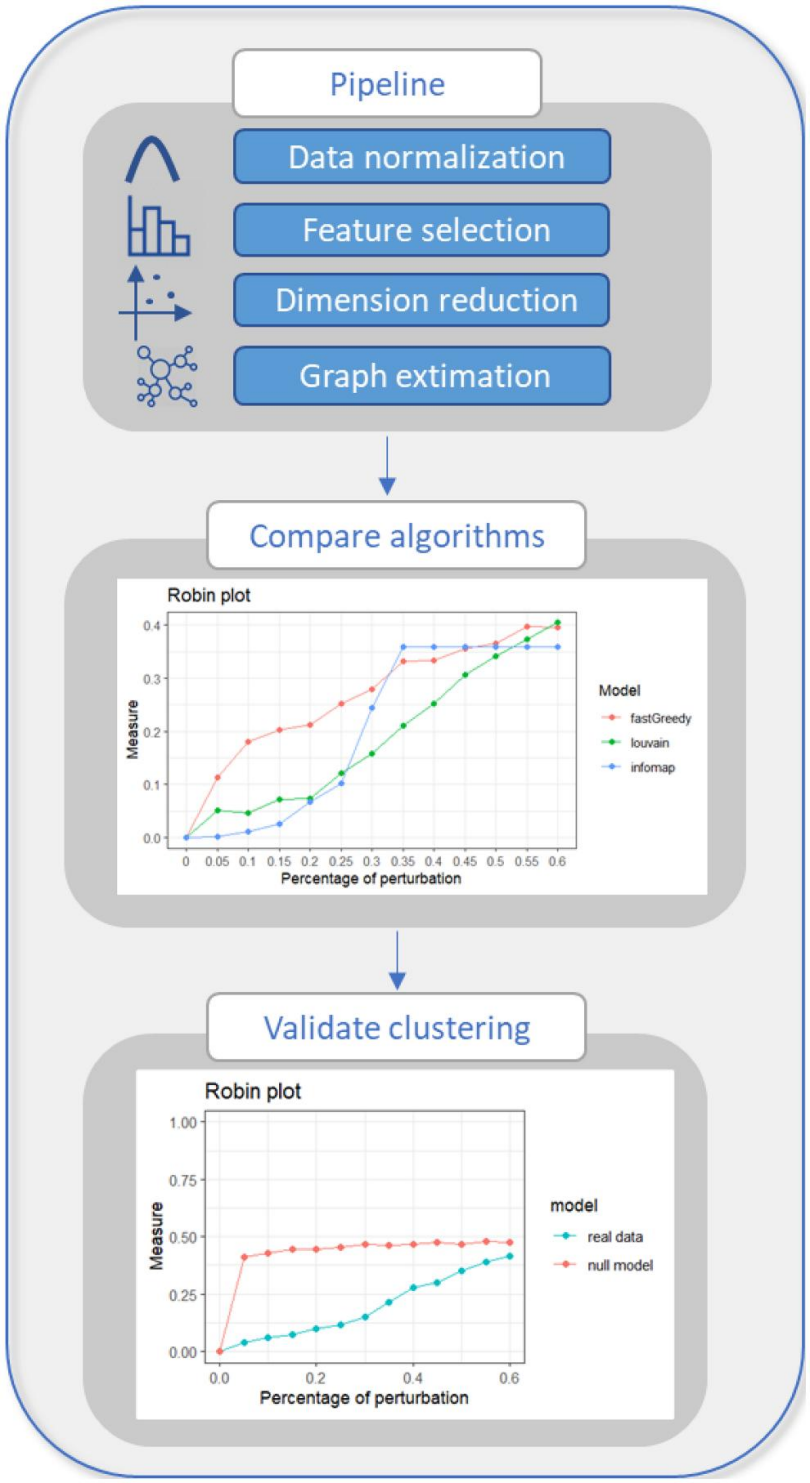
The workflow of single-cell analysis pipeline for clustering validation.

Given a network of interest, represented as the graph produced by a single-cell pipeline, the two procedures can be applied as follows (see Fig. 1). The first, implemented in the function robinCompare, is designed to compare various community detection algorithms to identify the one that best fits the cell network. Once the optimal algorithm is identified, the second procedure, implemented in the function robinRobust, validates the stability of the detected communities. Specifically, it tests how the cell subgroups identified in the network of interest compare to those found in a corresponding random network. The curves generated by these two procedures are compared using the tests implemented in the functions robinGPtest and robinFDAtest, along with the AUC analysis.

Our method not only evaluates the reliability of cell clustering but also aids in selecting the most suitable community detection algorithm for the cell network, by analyzing the graph structure independently of biological data. The core idea is to assess clusters based on the robustness of the underlying network, emphasizing network stability to identify the most reliable cell clustering solution. This approach is adaptable to various levels of optimization, enabling the selection of community detection algorithm parameters, such as the resolution parameter, the identification of the algorithm that best fits the cell network, and ultimately the choice of the most effective pipeline. Specifically, given a cell graph, robinCompare can generate multiple stability measure curves by varying the input parameters of a given community detection algorithm. The optimal parameters are those corresponding to the curve with the lowest AUC. Similarly, different community detection algorithms can be compared once their optimal parameters have been selected by applying them to the same cell graph for a systematic evaluation of clustering performance and identifying the best algorithm for that network. Finally, it is possible to compare clustering results across different single-cell analysis pipelines, such as Seurat and OSCA, to determine the pipeline that produces the most robust cell clustering (example in Fig. 3, available as supplementary data at *Bioinformatics Advances* online).

### 2.3 Computational aspects

robin2 introduces several novel features to enhance functionality and performance. To scale computations for high-dimensional datasets such as single-cell data, we have integrated a parallelized computational framework based on the bplapply function from the BiocParallel package, which ensures platform independence. This parallelization, applied to the percentage of perturbation, has been implemented in both robinCompare and robinRobust functions, significantly accelerating the computation of all steps associated with a given perturbation. (Table 1, Table 1, available as supplementary data at *Bioinformatics Advances* online, Figs 1 and 2, available as supplementary data at *Bioinformatics Advances* online). In Table 1, we present the reduction in execution times achieved by using parallelization with 12 cores compared to a single-core sequential algorithm in the previous version of robin. Each row of Table 1 corresponds to an increasing number of nodes and edges sampled from the dataset used in the Application section, highlighting a 9-fold improvement in execution time.

**Table 1.** Time performance for robinCompare across networks of different dimensions.^a^

Ncells	Edges	1 Core	12 Cores
580	17 429	0:0:15	0:0:3
2900	86 796	0:5:9	0:0:43
5800	189 771	0:14:49	0:1:49
8700	307 048	0:32:42	0:3:24
11 600	419 723	0:45:41	0:4:59
14 500	541 615	1:7:42	0:7:2
17 400	656 453	1:26:36	0:9:24
20 300	770 781	1:53:45	0:12:25
23 200	893 303	2:18:19	0:15:10
26 100	1 013 844	2:54:14	0:19:34
29 000	1 129 064	3:32:8	0:24:7

a Showing how the parallelization speeds up the computations by 9-fold.

To enhance robin’s interoperability with igraph, we have provided comprehensive support for all arguments across all clustering methods available in igraph and additionally the support for the previously missing Leiden algorithm. Our implementation enables users not only to compare different algorithms but also to evaluate the same algorithm under varying parameter settings, providing greater flexibility in performance assessment. The updated package also benefits from a dedicated robin class, which enables efficient storage of intermediate computations and final results. Additionally, this class ensures compatibility with the R base plotting interface, which streamlines result visualization. Lastly, we have introduced the plotMultiCompare function to improve comparative analysis. This function accepts multiple robin objects as input and generates a single comprehensive graph that summarizes the results in multiple methods (example in Fig. 5, available as supplementary data at *Bioinformatics Advances* online).

### 2.4 Application

The proposed method was applied to two datasets from different species: a subset of the Tabula Muris dataset (Tabula 2018) derived from mouse tissues and human Peripheral Blood Mononuclear Cells (PBMC) from the singleCellMultimodal Bioconductor package (Eckenrode *et al.* 2023). For the Tabula Muris, the data analysis was performed using the Seurat pipeline, detailed at https://satijalab.org/seurat/articles/pbmc3k_tutorial.html. The cell graph was constructed using the shared nearest neighbor algorithm in the reduced dimensional space as explained above. We applied robin2 to select an optimal community detection method that best approximates cell subtypes. Specifically, robin2 was used to compare clustering results from different community detection algorithms. The steps for the comparison and validation of clustering are summarized in Fig. 1.

The graph generated by Seurat consisted of 7986 cells and 279 713 edges (Table 2, available as supplementary data at *Bioinformatics Advances* online). Four community detection algorithms were applied to this graph obtaining different numbers of clusters (Fig. 4, available as supplementary data at *Bioinformatics Advances* online). To identify the clustering method that best approximates cell subtypes, robin2 was utilized with the robinCompare function (Comparison step of the Fig. 1). The results indicated that Louvain produced the most stable clustering, as evidenced by the lowest curve, followed closely by Walktrap, while the other algorithms exhibited higher variation (Fig. 5, available as supplementary data at *Bioinformatics Advances* online).

**Table 2.** VI between cell type and clusters.

Algorithms	*VI*
Louvain	1.05
Walktrap	1.07
Fast Greedy	1.14
Infomap	1.54
Label Propagation	1.61

The robustness (Validation step of the Fig. 1) of the Louvain algorithm was evaluated using the robinRobust function, comparing the communities detected in the real graph with those generated by a random graph based on a configuration model. The differences between the two resulting curves were also tested. The robinFDATest yielded all statistically significant *P*-values, and the Bayes Factor from robinGPTest exceeded 100, providing strong evidence that the two curves originate from distinct processes (Fig. 6, available as supplementary data at *Bioinformatics Advances* online). These results confirm the statistical significance of the communities detected by Louvain.

To evaluate how well the identified clusters (Fig. 7, available as supplementary data at *Bioinformatics Advances* online) approximated cell subtypes, the percentage of overlap between Louvain clusters and cell subtypes was calculated (Fig. 8, available as supplementary data at *Bioinformatics Advances* online). The analysis revealed that most clusters corresponded to a single subtype, aligning with the goal of scRNA-seq analysis. Some clusters contained more than one subtype, but these subtypes were biologically similar. For instance, Cluster *C27* included early pro-B cells, late pro-B cells, and Fraction A pre-pro B cells, while Cluster *C22* contained various monocyte types. These observations further support the conclusion that Louvain effectively approximates the cell subtypes within this network.

To assess the performance of the robin2 method, the ground truth of cell subtypes was compared to the partitions generated by each algorithm using the Variation of Information (VI) stability measure. As shown in Table 2, the VI measure corroborated the findings of the robin2 analysis, which were obtained without prior knowledge of the ground-truth. The results indicated that the Louvain and Walktrap algorithms were the most effective, producing clusters closely aligned with the ground truth. In contrast, Label Propagation demonstrated the poorest performance among the algorithms applied to this network (Fig. 5, available as supplementary data at *Bioinformatics Advances* online).

The human PBMC dataset was analyzed following the Bioconductor Orchestrating Single-Cell Analysis (OSCA) guidelines. We applied six community detection algorithms to the graph generated by OSCA (10 032 cells and 2 242 251 edges), obtaining different numbers of clusters.

To compare and validate the various community detection methods, we applied the same procedure described above. The most stable algorithm was Leiden, which identified 11 clusters (see Fig. 9, available as supplementary data at *Bioinformatics Advances* online). To assess the biological relevance of these findings, we annotated the communities using SingleR (Aran *et al.* 2019), with the MonacoImmuneData dataset from the celldex Bioconductor package as the reference. We found a strong correlation with the ground-truth cell types (see Fig. 10, available as supplementary data at *Bioinformatics Advances* online).

Furthermore, our pipeline can transfer the obtained clusters to either a Seurat or SingleCellExperiment object and perform differential expression analysis to identify marker genes defining each cell subtype.

Detailed analysis pipelines for both datasets, including the integration of robin2 within both Seurat and OSCA frameworks, are publicly available at https://drighelli.github.io/scrobinv2/index.html.

## 3 Discussion

In this work, we have demonstrated how to apply robin2 to scRNAseq data, where the clustering step is a fundamental aspect in any dataset analysis for the identification of the cell types. Indeed, with the aid of our methodology, we are now able firstly to compare multiple clustering algorithms and then verify the robustness of the obtained communities. Additionally, we have enhanced robin computational efficiency by several orders of magnitude, making it practical also for big single-cell datasets. We achieved this by implementing in robin2 the parallelization of computations over the percentage of the rewiring strategy, which in our method is equal to 12. To improve the statistical robustness of the test, each percentage of perturbation consists of 10 replicates. To further speed up the computation, future improvements could potentially be achieved by parallelizing the computations for individual replicates as well. Performances in terms of running time are now acceptable even with large datasets.

Finally, we demonstrated how to select the optimal community detection method that best approximates cell subtypes by comparing different community detection algorithms. However, our method offers greater potential and can also be used to optimize parameter choices, such as the resolution parameter, within the same algorithm. Furthermore, the application of our method enables the comparison of different single-cell analysis pipelines, such as Seurat, OSCA, and others, through the generated graphs, thereby aiding in the more precise definition of cell subtypes.

## Supplementary Material

vbaf184_Supplementary_Data
