# Supplementary material for: robin2: accelerating single-cell data clustering evaluation
Source: Bioinform Adv. 2025 Aug 6;5(1):vbaf184. doi: 10.1093/bioadv/vbaf184 (PMC12341891; doi:10.1093/bioadv/vbaf184)
Supplement: vbaf184_Supplementary_Data [file vbaf184_supplementary_data.pdf]

Supplementary Material for *robin2*: Accelerating Single Cell Data Clustering Evaluation

Valeria Policastro[1,4,§], Dario Righelli[2,§], Luisa Cutillo[3, ξ], and Annamaria Carissimo[4,ξ, *]

[1]Department of Political Science, University of Naples Federico II, Via Leopoldo Rodinò 22, 80133, Naples, Italy
[2]Department of Electrical Engineering and Information Technology, University of Naples Federico II, Via Claudio 21, 80125, Naples, Italy
[3]School of Mathematics, University of Leeds, Woodhouse, LS2 9JT, Leeds, UK
[4]Consiglio Nazionale delle Ricerche (CNR), via Pietro Castellino, 111, 80131, Naples, Italy

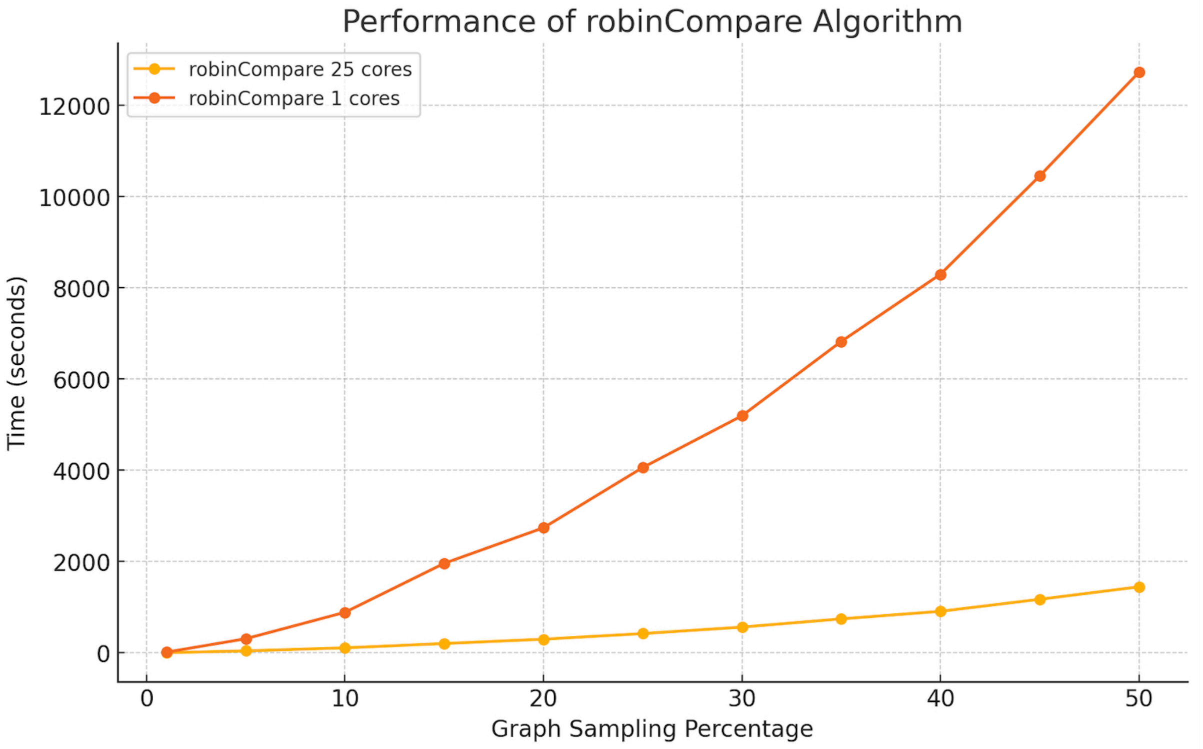

Figure S1: Performance of `robinCompare` using 1 and 12 cores.

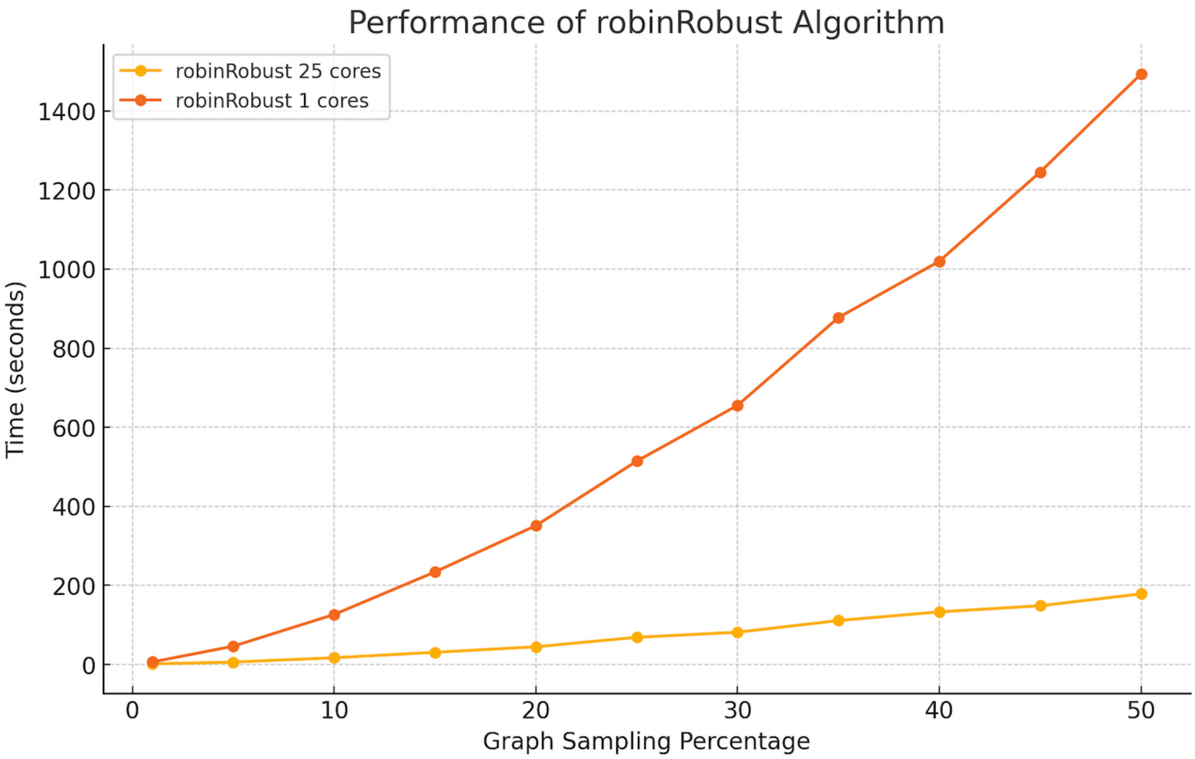

Figure S2: Performance of `robinRobust` using 1 and 12 cores.

Percentage	Ncells	Edges	Algorithm	Cores	Hours	Minutes	Seconds
1%	580	17429	`robinCompare`	12	0	0	2.43
1%	580	17429	`robinCompare`	1	0	0	14.64
1%	580	17429	`robinRobust`	12	0	0	1.73
1%	580	17429	`robinRobust`	1	0	0	6.74
5%	2900	86796	`robinCompare`	12	0	0	42.94
5%	2900	86796	`robinCompare`	1	0	5	8.94
5%	2900	86796	`robinRobust`	12	0	0	6.33
5%	2900	86796	`robinRobust`	1	0	0	46.46
10%	5800	189771	`robinCompare`	12	0	1	49.22
10%	5800	189771	`robinCompare`	1	0	14	48.85
10%	5800	189771	`robinRobust`	12	0	0	17.23
10%	5800	189771	`robinRobust`	1	0	2	6.63
15%	8700	307048	`robinCompare`	12	0	3	24.09
15%	8700	307048	`robinCompare`	1	0	32	41.86
15%	8700	307048	`robinRobust`	12	0	0	31.03
15%	8700	307048	`robinRobust`	1	0	3	54.26
20%	11600	419723	`robinCompare`	12	0	4	58.84
20%	11600	419723	`robinCompare`	1	0	45	41.24
20%	11600	419723	`robinRobust`	12	0	0	44.92
20%	11600	419723	`robinRobust`	1	0	5	51.25
25%	14500	541615	`robinCompare`	12	0	7	2.14
25%	14500	541615	`robinCompare`	1	1	7	41.65
25%	14500	541615	`robinRobust`	12	0	1	8.97
25%	14500	541615	`robinRobust`	1	0	8	34.31
30%	17400	656453	`robinCompare`	12	0	9	24.08
30%	17400	656453	`robinCompare`	1	1	26	36.35
30%	17400	656453	`robinRobust`	12	0	1	21.76
30%	17400	656453	`robinRobust`	1	0	10	55.40
35%	20300	770781	`robinCompare`	12	0	12	24.79
35%	20300	770781	`robinCompare`	1	1	53	45.06
35%	20300	770781	`robinRobust`	12	0	1	51.31
35%	20300	770781	`robinRobust`	1	0	14	37.30
40%	23200	893303	`robinCompare`	12	0	15	9.90
40%	23200	893303	`robinCompare`	1	2	18	18.95
40%	23200	893303	`robinRobust`	12	0	2	13.25
40%	23200	893303	`robinRobust`	1	0	16	59.66
45%	26100	1013844	`robinCompare`	12	0	19	33.79
45%	26100	1013844	`robinCompare`	1	2	54	13.74
45%	26100	1013844	`robinRobust`	12	0	2	28.70
45%	26100	1013844	`robinRobust`	1	0	20	45.26
50%	29000	1129064	`robinCompare`	12	0	24	6.72
50%	29000	1129064	`robinCompare`	1	3	32	8.02
50%	29000	1129064	`robinRobust`	12	0	2	58.78
50%	29000	1129064	`robinRobust`	1	0	24	52.98

Table S1: Performance of `robinCompare` and `robinRobust` across different sampling percentages.

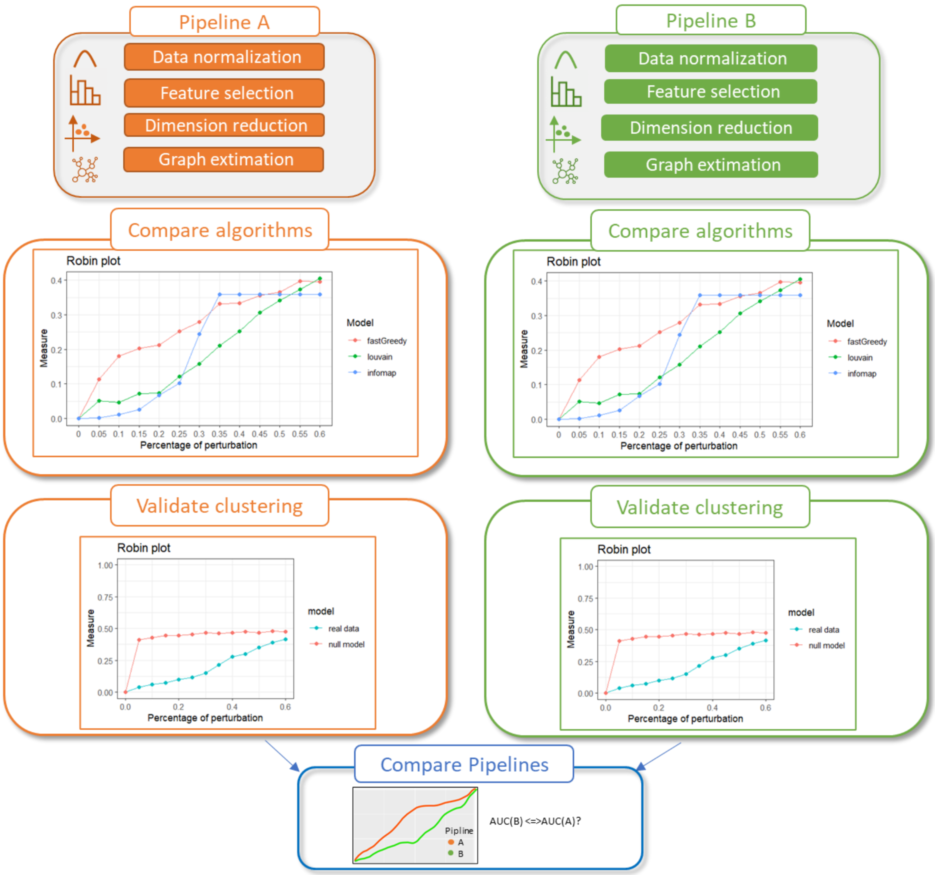

Figure S3: Workflow for the analysis, comparison, and validation of clustering results in scRNA-seq data using two different pipelines.

V	***E***	***transitiv.***	***diameter***	***modularity***	***density***
7986	279713	0.586479	14	0.905718	0.008773

Table S2: Measure of the Tabula Muris graph taking a sample of 15% of the overall dataset. Modularity is calculated on Louvain communities. (The acronym V stands for vertices, E stands for edges and *transitiv.* stands for transitivity).

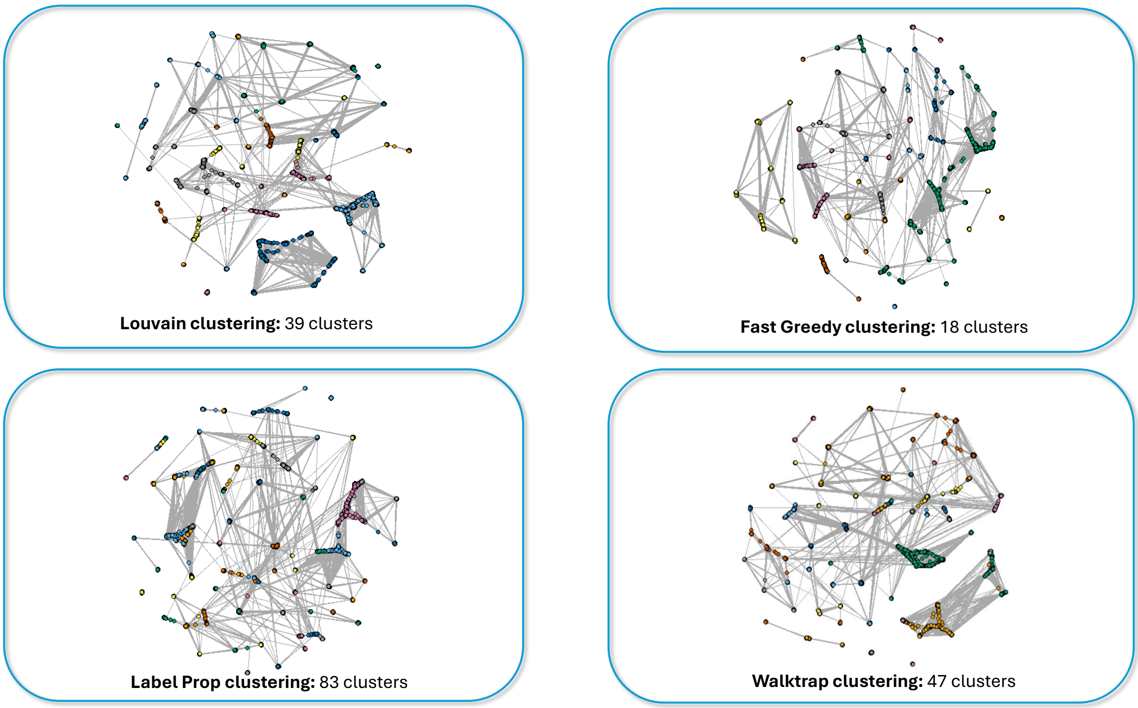

Figure S4: Community structures identified by different detection algorithms on the Tabula Muris network: Louvain, Fast Greedy, Label Propagation, and Walktrap.

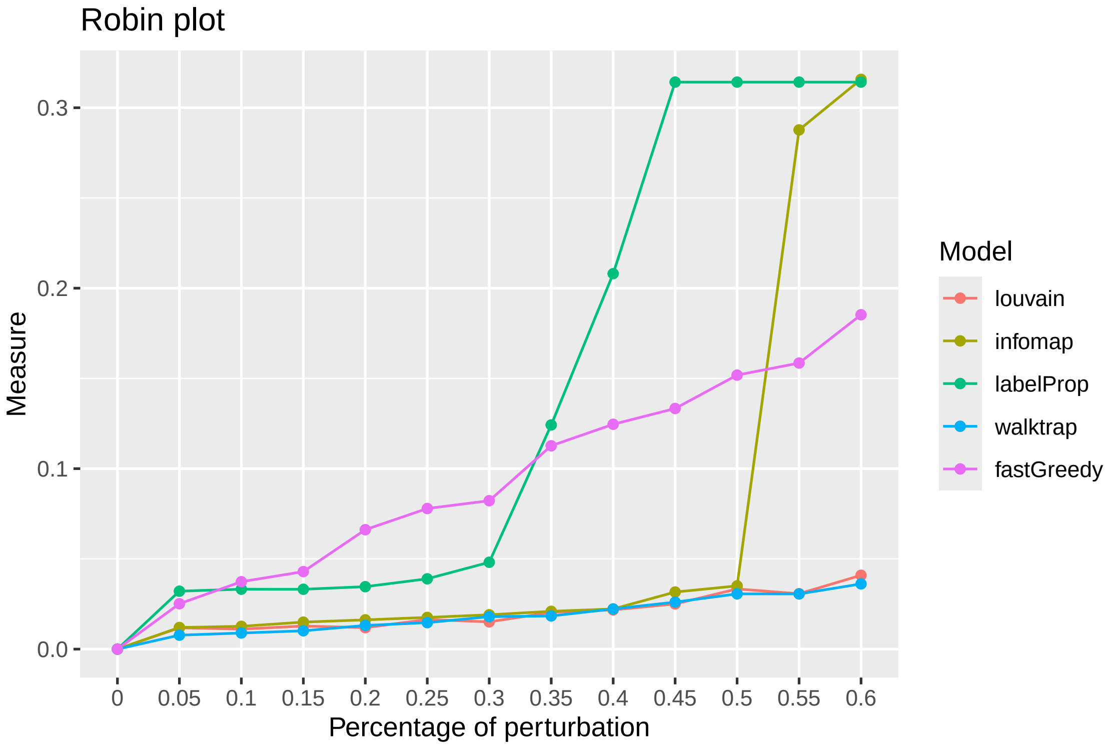

Figure S5: Comparison of different community detection algorithms on the Tabula Muris dataset, generated using the `robinCompare` method and the `plotMultiCompare` function.

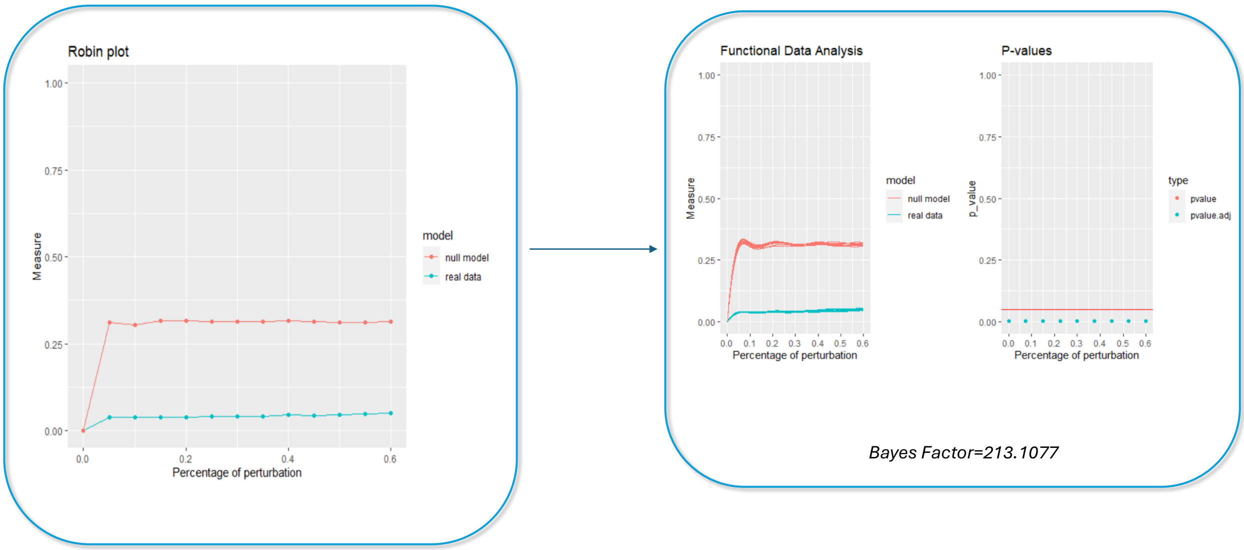

Figure S6: Validation of the Louvain community detection method. Left panel: Output plot of the `robinRobust` function applied to the Louvain algorithm. Right panel: Results of two tests: on the left, the FDA test represented by the p-value plot, and on the right, the Bayes factor.

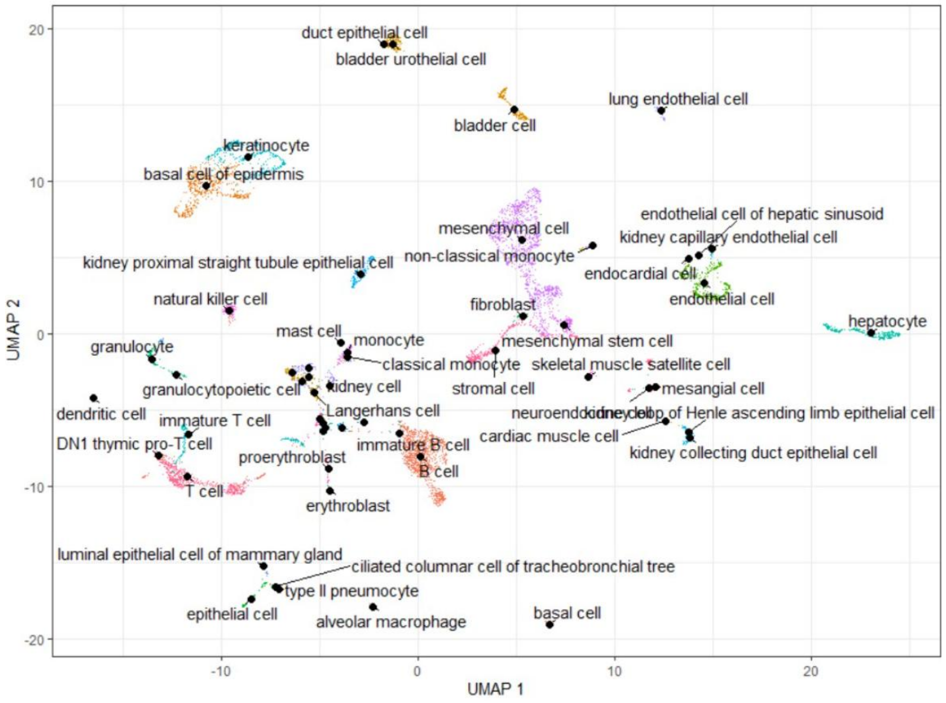

Figure S7: UMAP representation of Louvain communities and cell type labels in the Tabula Muris dataset.

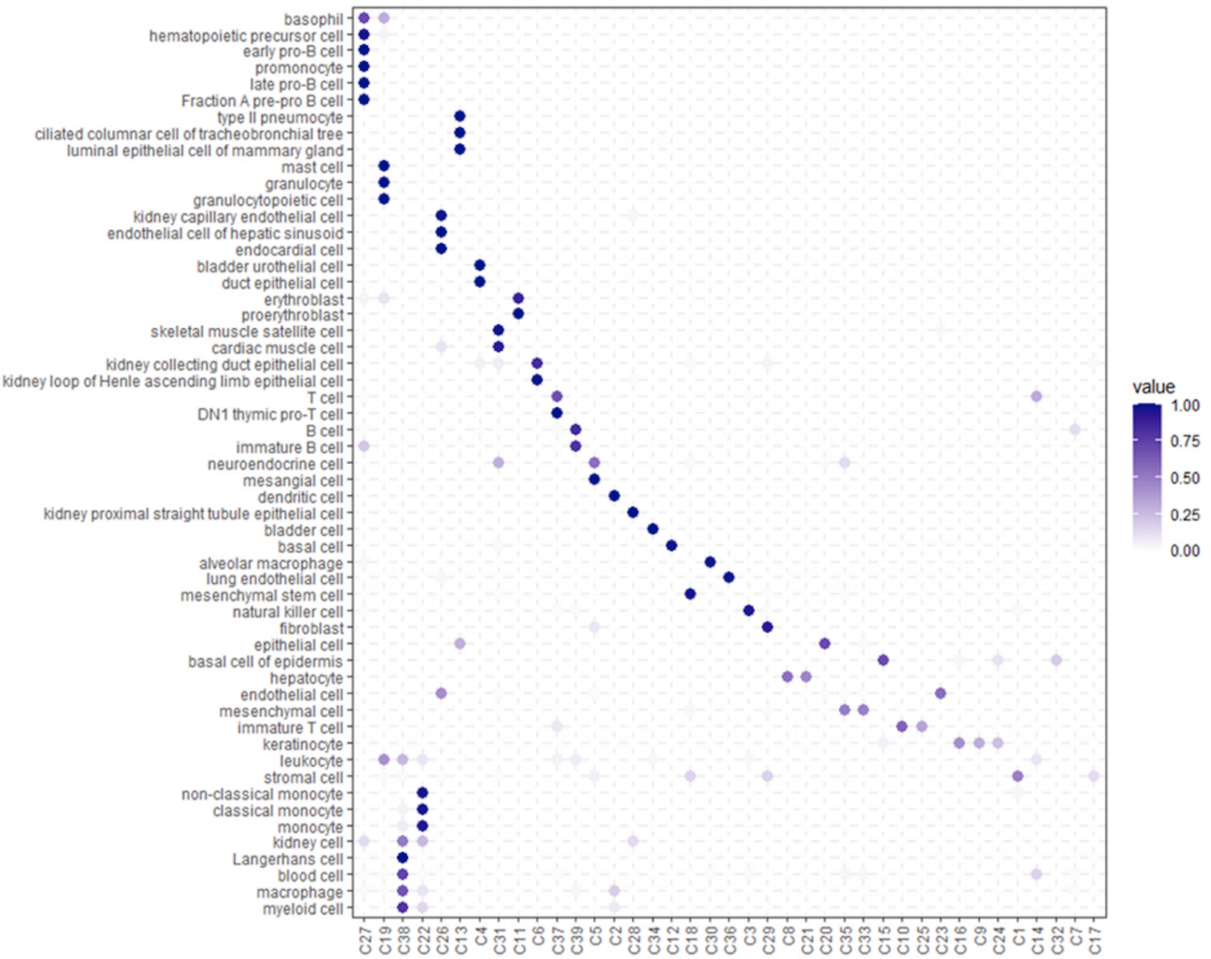

Figure S8: Dotplot showing the percentage distribution of cell subtypes across clusters, based on the Tabula Muris dataset. The x-axis represents the Louvain clusters, while the y-axis corresponds to the cell subtypes from the metadata. Each row sums to 1, indicating the proportion of each cell subtype within a given cluster. The plot highlights that each cell subtype is predominantly associated with a specific cluster.

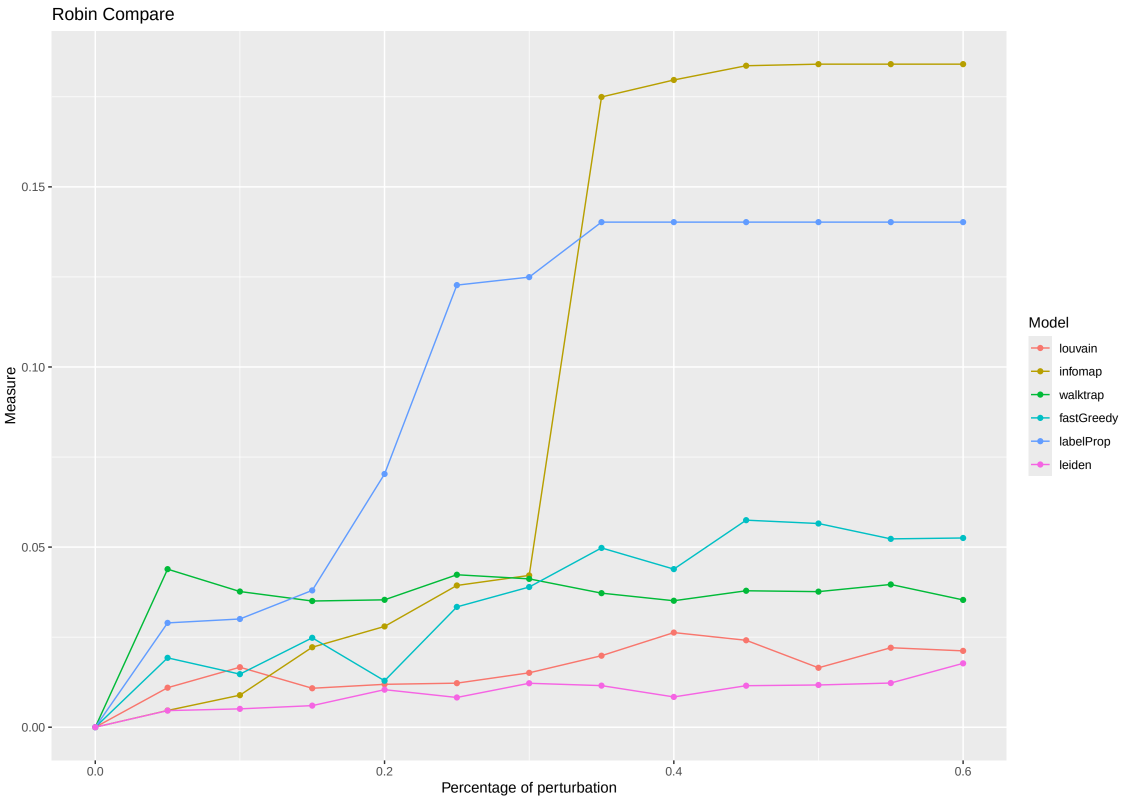

Figure S9: Comparison of different community detection algorithms on the PBMC dataset, generated using the `robinCompare` method and the `plotMultiCompare` function.

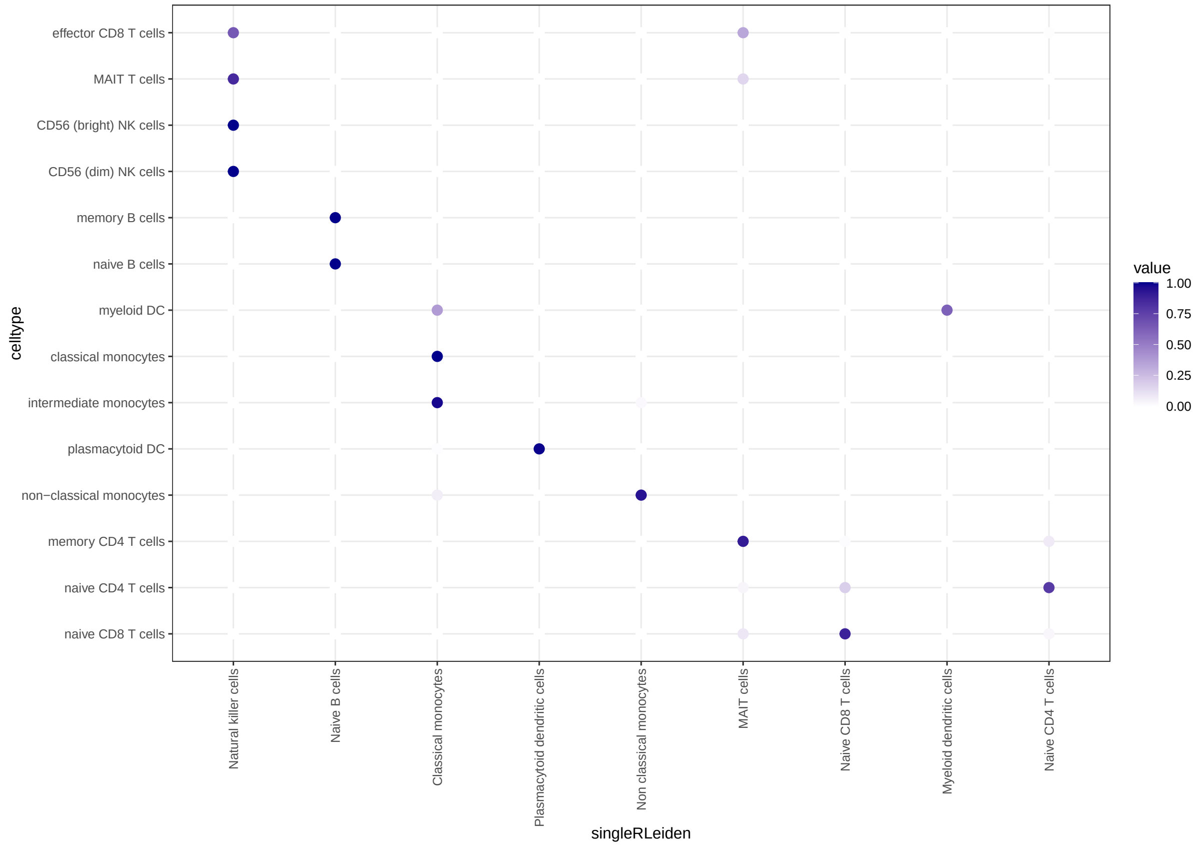

Figure S10: Dotplot showing the percentage distribution of cell subtypes across clusters, based on the PBMC dataset. The x-axis represents the Leiden clusters annotated with SingleR, while the y-axis corresponds to the cell subtypes from the metadata. Each row sums to 1, indicating the proportion of each cell subtype within a given cluster. The plot highlights that each cell subtype is predominantly associated with a specific cluster.
